# Poisoning by Arsenic

**Published:** 1841-03

**Authors:** 


### Selections from American and Foreign Journals.

---

*Poisoning by Arsenic. Meetings at the Faculty of Medicine in Paris.*—We have seldom attended meetings of a more imposing nature than those recently held in Paris upon a most important question of medical jurisprudence, which has arisen out of the celebrated case of Lafarge, who was poisoned by his wife. The members of the Academies of Sciences and of Medicine, together with the élite of the practitioners resident in the French metropolis, had received invitations from M. Orfila, to witness the exposition of his methods of detecting poison by arsenic and antimonial salts: as also a demonstration of the successful mode of treating poisoned animals, by bleeding and diluent diuretics, after evacuating the poison from the primæ viæ.

Nine chemists, employed on the trial of the female culprit at the assizes of Tulle, had failed to detect arsenic in the organs of the poisoned man; M. Orfila, with Messrs. Bussy and Oliver d'Angers, who were subsequently consulted, succeeded in extracting the arsenic, which they exhibited to the court, in the form of metallic spots on porcelain capsules, and thus dissipated all doubt on the subject.

The chemists previously employed were men of eminence, theoretically acquainted with the mode of detecting arsenic, but not practically experienced in this delicate branch of chemical manipulation; and as thousands of medical practitioners would find themselves in the same predicament, to the great injury of the interests of justice, M. Orfila has rendered a great service to the cause of science, by publicly demonstrating the proper path to be pursued, more especially as he has carefully elucidated the errors likely to be committed, so as to produce failure.

Another question setted at these meetings, relates to the suposed impurity of tests employed in detecting arsenic. It is quite obvious that if the zinc, sulphuric acid, and water employed in the process, themselves contain the metal, we cannot be certain of its existence in the organs submitted to experiment with those tests. All who know M. Orfila are aware of his extreme caution to ascertain the purity of his tests at the moment of using them. When the hydrogen gas has been produced from these substances, nothing can be more easy than to suffer Marsh's apparatus which contains them to work for a quarter of an hour; and if, during that period, the gas inflamed at its exit from a very fine pointed tube, bent at right angles from the bottle, deposits no arsenical spots on a porcelain capsule properly directed to its point, we may safely infer that it contains no portion of the metal in question; and if, at the expiration of that period, we add a portion of the suspected matter, prepared as hereinafter to be described, and the arsenical spots can then be made to appear on the capsule, the existence of the poison is fairly demonstrated.

A most absurd and calumnious insinuation has been circulated in relation to the case of Lafarge, that Orfila employed a nitrate of potash which might have been impure from admixture with arsenic. The learned dean, in answer to this, declares that he has examined two hundred different specimens of the commonest saltpetre of commerce, without ever finding them to contain a particle of arsenic.

The main object of the meetings was to demonstrate, by experiments on animals, 1, that arsenious acid and tartarized antimony, either introduced into the primæ viæ, or placed under the subcutaneous cellular tissue, are absorbed, mixed with the circulating fluid, and thus conveyed into all the organs of the animal body. 2. That they sojourn for a certain time in the visceaa and muscles, where their presence may be demonstrated by Marsh's apparatus for the production of hydrogen, which dissolves the metallic salt, and again deposits it on a cold surface by combustion of the gas with certain precautions; but that at a very early period after the ingestion of the poison, *a part* of what has been absorbed is eliminated by the urine, and consequently has abandoned the animal tissue. 3. That this elimination, which is much more rapid for the emetic tartar than the arsenious acid, continues for several days, until the tissues are completely freed of the poison. 4. That on this account it is *indispensable*, in the treatment of poisoning from these venomous substances, to promote the *secretion of urine*, and to abstract the deleterious blood. 5. That, in the majority of cases, it is possible to distinguish whether the arsenious acid and the tartarized antimony, which are extracted from a dead body, had been administered during life, or by cadaveric imbibition after death—this last might occur in cases of poison being put into the stomach or rectum, of a person deceased, in order to raise a false accusation of poisoning against an innocent individual. 6. That the best mode of detecting minute portions of these mineral poisons, when absorbed into the organs, consists in destroying the greater part or the whole of the organic matters, by first drying them in a porcelain capsule over a fire, and then carbonizing them, either by concentrated azotic acid, or by deflagration with azote of potash; after which the residuum, if introduced into Marsh's apparatus, modified for the production of hydrogen gas, would come over as metallized gas, if the liquor should contain the metal. 7. That it is always easy to distinguish arsenic from antimony in the form of spots, when deposited from the metallized gas on the porcelain capsule; and that we may be assured these spots neither proceed from the tests employed, nor from the apparatus itself, by suffering the latter to work for a certain time before we add the suspected matter, and by previously making trial of the inflamed gas upon the porcelain capsule destined to receive the metallic precipitate, if any should exist. 8. That the *bones* of man and several animals contain an arsenical compound insoluble in water, and which differs from the arsenic absorbed by the organs. 9. That we may extract from human muscles a matter which M. Orfila considers to contain an *infinitely minute* proportion of arsenic, sulphur, and an organic substance; which compound differs essentially from the arsenical spots produced from the organs, or the urine, in case poisoning. 10. That we find in the earth of certain churchyards *infinitely minute* quantities of arsenic *insoluble in boiling water*. 11. Finally, that in legal medicine we may easily avoid any error which the presence of these minute portions of arsenic in bones, muscles, and the soil of churchyards, seems at first sight likely to produce.

The meetings for the above demonstrations were held on Oct. 25, 26, Nov. 1, 2. The experiments on the animals were performed at a preliminary meeting at ten o'clock in the following order; and the results were verified, and other collateral experiments were performed, at two.

First. The œsophagus was cut down upon in the neck of a dog, and tied; the ligature was to be cut away after twenty-eight hours, to show that the animal would eat and drink as usual; from which we might infer, that in the poisoned dogs, with ligature on the œsophagus, to prevent the vomiting of the poison, the animal was not injured by the ligature. All this has been verified.

Second dog. The penis was tied, in order that its urine might be collected and compared with the same fluid in a poisoned dog.

Third dog. Hanged, in order that poison might be introduced into the stomach, and there left for eight days, to show that the adjacent organs would *imbibe* it from the stomach.

Fourth dog. Twelve grains of arsenic in solution were introduced into the stomach by a glass funnel, through an incision in the œsophagus. The dog was to die before two o'clock, the hour appointed for the second meeting, which was verified.

Fifth dog. Instead of arsenic a solution of twelve grains of tartarized antimony was poured into the stomach, as in the last case. Death, as in the last case.

Sixth dog. Three grains of arsenic were placed in the subcutaneous cellular tissue of the thigh. Death predicted, and realized in twenty-four hours.

Seventh dog. Tartarized antimony in the same dose (three grains) was put into the subcutaneous cellular tissue. Death in twenty-four hours, as in the former case.

In the last four animals the penis was tied, in order to preserve the urine; which fluid should be the first to be examined, either in case of suspected poison during life, or after death, whenever it can be obtained. To neglect this in either case would be a capital blunder. During life it may be the only matter within the reach of the practitioner that can be tested; after death it may happen that all the metallic poison has been eliminated from the organs, but may still be in the urine. The first thing, therefore, to be done in post mortem examinations of persons suspected to be poisoned, is to secure the urine, if the bladder should contain any; nay, in one of the experiments at the meeting of the 26th, on the dog poisoned by three grains of arsenic, which had been left for twenty-four hours in the subcutaneous cellular membrane of the thigh, no urine was found, and it could not have escaped from the urethra, in consequence of the penis having been tied; *yet the washing of the inner coat of the bladder detached a portion of arsenic from its surface*, and the liquor introduced into Marsh's apparatus deposited the metal in the usual way.

The urine of the healthy dog was found to contain no arsenic. This and another experiment on the healthy liver was performed with the view of imposing silence upon cavillers who are seeking to obtain popularity as the patrons of criminals, by insinuating that *normal* arsenic is to be found in the human body, which, with certain limitation as to parts, is true; but is sufficient for medico-legal purposes to show that no normal arsenic is contained either in the liver or in the urine, because in case of poisoning we need not go beyond those parts for the detection of the absorbed metal.

*Detection of Arsenic and Antimony in the Urine.*—These metals, which are soluble in hydrogen, so as to form arseniated or antimoniated hydrogen respectively, may be easily expected to precipitate in a metallic form, on the combustion of the gas which holds them in solution; but that precipitation would be lost, unless the point of the gaseous flame were directed, *secundem artem*, on some *cold* surface capable of receiving the deposit, and the necessity of its being a cold surface, such as a white porcelain capsule, arises, as the case of arsenic, from the volatility of that metal by heat. The experiment, therefore, might be defeated, by using so large a flame as to heat the surface; to prevent this, the point of the tube from which it emanates must be so fine, as to allow a flame not larger than about one-sixth of an inch in length; the suspected urine is to be put into Marsh's bottle with zinc, sulphuric acid and water, for the production of hydrogen; if it contain arsenic, the inflamed gas, at the point of a tube inserted into the cork of the bottle, and bent nearly at right angles, will deposit the metal on the porcelain capsule in the form of yellowish brilliant spots, soluble in nitric acid; the dried salt thus produced being convertible into red arsenate of silver by nitrate of silver, if a sufficient quantity of nitric acid had been used; the arsenical spots may be further distinguished from the antimonial by its volatility at the flame-point of pure hydrogen, to say nothing of the difference of aspect which should be learned by actual experiment.

The detection of antimony in urine cannot be effected without evaporating the liquid to dryness, and carbonizing the residuum by boiling in azotic acid, or deflagrating with saltpetre; which latter, it may be remarked, cannot be depended upon for the carbonization of arsenical urine, inasmuch as that metal might be volatilized by the intensity of the deflagration.

After the carbonization of the residuum of antimonial urine, the tartaric acid, which forms the salt of the contained metal, has been decomposd, and the antimony is left in a form insoluble in water, until a portion of chlorhydric acid be added. In this state the coal is to be boiled for two or three hours in distilled water, the decoction is put into the hydrogen bottle, with zinc and diluted sulphuric acid, and the spots of antimony are speedily precipitated on the porcelain capsule from the inflamed metallized gas.

In treating urine, the operator is liable to be incommoded by froth and albuminous matter floating upon the liquor within the apparatus. To remove this inconvenience when it occurs, let the whole be put into a glass funnel, until the frothy matter be settled on the surface; in which case the clear liquor can be easily separated, and returned into the apparatus.

It will be observed, that the arsenical and antimonial spots are both soluble in nitric acid, and differ little in appearance until after the application of heat, which, by supplying an additional dose of oxygen, converts the arsenious acid into arsenic acid; and this, again, can be decomposed by nitrate of silver, forming the arseniate of silver of a brick-dust color. No such change is produced in the antimonial salt; and it may be well to state, thut the spots produced from the *normal* arsenic of muscular fibres differ in color from the *absorbed* arsenic in poisoning. The normal spots are an *opaque white, insoluble in cold nitric acid*, but soluble in the *boiling* acid. They are not convertible into arseniate of silver of a brick-dust color by *neutral* nitate of silver, after their solution in *boiling* nitric acid has been evaporated to dryness by heat, and they are volatile.

In order to show these spots of *normal* arsenic, which M. Orfila remarks are in *infinitely minute* proportion, the learned professor boiled a quantity of human flesh in distilled water, with potash, for five hours; the decoction strained and evaporated to dryness, was carbonized by azotic acid. It was then boiled for half an hour in distilled water, and put into the apparatus in presence of the assembly. In a few minutes the opaque white spots were received on a porcelain plate, and handed round the amphitheatre.

To contrast the *normal* arsenic with that produced in the human flesh by absorption from the stomach, a porcelain capsule was also exhibited with numerous arsenical spots, collected from the arm of the assassin Soufflot, who poisoned himself on receiving sentence of death.

M. Orfila has made repeated experiments on the urine of patients who had taken emetic tartar for inflammation of the lungs. The antimony in such cases can always be detected by Marsh's apparatus. The spots produced are not easily volatized; and the nitrate of antimony formed by dissolving the spot in nitric acid, and evaporated to dryness by heat, is not reddened by nitrate of silver, like arsenic.

*Detection of Arsenic in the Liver of one of the poisoned Dogs.*—The dog poisoned by twelve grains of arsenic in solution poured into the stomach, the œsophagus having been tied to prevent vomiting, was in *articulo mortis* at the expiration of four hours. His liver was taken out, and M. Orfila directed attention to the fact, that the stomach and intestines had not been perforated so as to give issue to the poison. If, therefore, arsenic was found in the liver, its presence could only be accounted for by absorption in the regular way, through the circulation. One fourth of the liver was cut into minute portions, and dried in a porcelain capsule over a charcoal fire, then carbonized by nitric acid, and boiled in distilled water. The decanted liquor was put into the hydrogen bottle, and in a few seconds the spots were deposited on the porcelain capsule.

The liver of the healthy dog treated in the same manner gave no trace of arsenic; and, as this result has been constantly witnessed, it may be inferred that the healthy liver contains no *normal* arsenic, although the metal in a modified form has been found in the human flesh and bones.

One of the experiments made upon three-fourths of the liver of the poisoned dog, produced fewer arsenical spots than that upon the fourth. This was owing to the carbonization having been effected by deflagration with nitrate of potash, which, from the intensity of its flame, had volatilized a portion of the metal. The nitrate of potash, therefore, is only to be adopted when the carbonization cannot be completed by azotic acid, in consequence of the parts having been more or less converted into *adipocire*, or, as M. Orfila says, saponified.

A caviller has publicly affirmed that arsenic may be developed in the soft parts by putrefaction, although it might not exist, or be discoverable, in the normal state. A paper was, therefore, addressed to M. Orfila, by one of the academicians, requesting that an experiment might be made on a putrid liver, in order to settle this question. At the meeting of Nov. 1, M. Orfila had provided a liver, which had been in the dissecting room of the faculty for twelve days, and was in an advanced state of putrefaction on the day of meeting. In order to afford a greater chance of collecting the arsenic, if any should exist, it was resolved to carbonize the whole of the liver, instead of taking the dried residuum of its decoction, as in the former experiment on the sound liver. The whole was, therefore, carbonized by nitric acid in the presence of the commission of the Academy of Sciences; which was not effected without much difficulty, in consequence of the change undergone by putrefaction. The mass was then boiled in distilled water, and the decoction was placed in Marsh's apparatus, in the presence of the audience. Not an atom of arsenic could be traced. M. Lepelletier, as one of the commission of the Academy, tried his hand in directing the flame of the hydrogen upon the porcelain plate; but nothing was procuced, excepting *a nnmber of white spots, which are furnished by animal matter.* As this experiment is not a solitary one, Mr. Orfila, maintains that *normal arsenic is not to be found in the liver, whether it be putrid or fresh.*

When the person poisoned by arsenic has taken emetic tartar for the purpose of producing vomiting, the blood, the organs and the urine will contain both the metals, which may be reproduced by Marsh's apparatus. The aspect of the mixed metals will vary according to the proportions in which they respectively exist. In order to illustrate this, M. Orfila exhibited the results of different proportions of the respective metallic solutions on a porcelain plate; from two drops of a concentrated solution of emetic tartar for instance, to two of arsenic, then 2 to 3, 2 to 4, &c., the arsenic being the increasing substance. Another series was formed out of larger quantities, beginning with 9 grains of arsenic to 9 of emetic tartar, then 9 to 10, 9 to 11, &c., the ascending series being the antimonial salt.—*Lancet. Boston Med. and Sur. Jour., Jan.* 13, 1841.

